# Magma diversity reflects recharge regime and thermal structure of the crust

**DOI:** 10.1038/s41598-020-68610-1

**Published:** 2020-07-17

**Authors:** Gregor Weber, Guy Simpson, Luca Caricchi

**Affiliations:** Department of Earth Sciences, University of Geneva, Rue des Maraîchers 13, 1205 Geneva, Switzerland

**Keywords:** Petrology, Volcanology

## Abstract

The chemistry of magmas erupted by volcanoes is a message from deep within the Earth’s crust, which if decrypted, can provide essential information on magmatic processes occurring at inaccessible depths. While some volcanoes are prone to erupt magmas of a wide compositional variety, others sample rather monotonous chemistries through time. Whether such differences are a consequence of physical filtering or reflect intrinsic properties of different magmatic systems remains unclear. Here we show, using thermal and petrological modelling, that magma flux and the thermal structure of the crust modulate diversity and temporal evolution of magma chemistry in mid to deep crustal reservoirs. Our analysis shows that constant rates of magma input leads to extractable magma compositions that tend to evolve from felsic to more mafic in time. Low magma injection rates into hot or deep crust produces less chemical variability of extractable magma compared to the injection of large batches in colder or shallower crust. Our calculations predict a correlation between magma fluxes and compositional diversity that resembles trends observed in volcanic deposits. Our approach allows retrieval of quantitative information about magma input and the thermal architecture of magmatic systems from the chemical diversity and temporal evolution of volcanic products.

## Introduction

**Figure 1:**
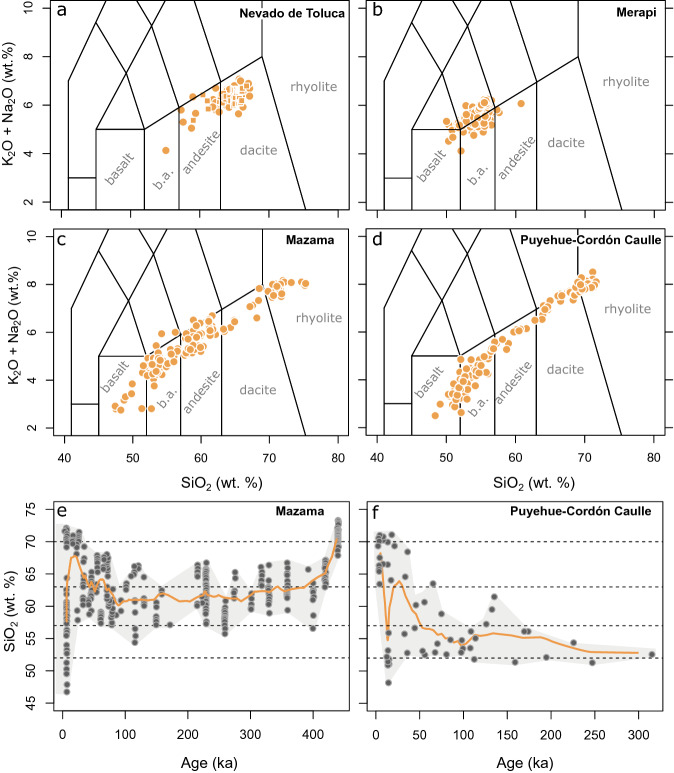
Differences in the geochemical variability of Arc volcanoes. Total alkali (K_2_O + Na_2_O wt%) versus silica (SiO_2_ wt%) plots with indicated compositional fields of whole rock data representing the long-term eruptive histories of well-studied volcanic systems. (**a**) Nevado de Toluca (Trans Mexican Volcanic Belt). (**b**) Merapi (Sunda Arc, Indonesia). (**c**) Mazama-Crater Lake (Cascades, Oregon). (**d**) Puyehue-Cordón Caulle (Southern Volcanic Zone, Chile). (**e**) SiO_2_ (wt%) versus age for Mazama modified from Ref.^6^. (**f**) SiO_2_ (wt%) of dated eruptive products for Puyehue-Cordón Caulle modified from Ref.^8^. Orange lines in (**e**) and (**f**) are moving averages, grey shading indicates range of compositions. Data was taken from the GEOROC database (https://georoc.mpch-mainz.gwdg.de/georoc/) and new whole rock analysis for Nevado de Toluca ("Methods" section, Supplementary table S3). The data exemplify that volcanic systems show large differences in the variability of erupted compositions.

Understanding the variability and temporal evolution of erupted magma chemistry is critical to quantify magmatic and ore forming processes^1–3^, and to anticipate the potential future activity of volcanoes^4^. Detailed age-resolved geochemical records show that some volcanic centres erupt a wide variety of magma compositions, while others produce restricted chemical diversity throughout their lifetime^5–12^ (Fig. 1). Additionally, for systems that sample a large variability of magma types, the composition and variety of erupted magmas change over time (Fig. 1e, f; Fig. S3). Differences in compositional diversity and temporal trends between individual volcanoes have been attributed to different mechanisms. The evolving rheological properties of the crustal rocks hosting magma reservoirs can modify the capacity of magmas to rise to the surface and erupt or accumulate at depth^13–16^, which may impact on compositional diversity. The physico-chemical properties of the magma itself are a first order control in this respect. Density or viscosity barriers may prevent magmas of specific chemistry from erupting, leading to erupted magmas with a rather monotonous composition^17,18^. Such barriers might be overcome by mixing of mafic and silicic compositions^10,19^ or develop in parallel with the construction and destruction of large volcanic edifices^20^. In these models, volcanoes are depicted as physical property filters that sample only a part of the compositional spectrum present in their plumbing system. It is not clear, however, why such filters would be effective in volcanoes with restricted chemical variability and not be as effective in systems where erupted magmas exhibit a variety of compositions. Alternatively, differences in the range of erupted magma compositions could reflect contrasting recharge regimes and thermal states of crustal magma reservoirs feeding volcanoes^21^. In this study we further test this hypothesis, exploring the thermochemical evolution of mid to deep crustal magmatic systems subjected to injection of hydrous basaltic magma.

### Thermal and petrological modelling

To establish the link between magma recharge rates and the evolution of extractable magma chemistries in mid crustal reservoirs, we developed a numerical model that couples heat transfer and phase petrology. The design of our model is motivated by previous numerical studies^22–25^, petrological findings^26–29^ and geophysical evidence^30–32^ that mantle-derived hydrous basalt crystallization at mid to lower crustal depth plays a key role in generating intermediate to silicic subduction-related magma. We do not simulate magma extraction and eruption, but we trace the evolution in the chemistry of potentially extractable magma (here defined as magma with < 50% crystallinity and all interstitial melt) over time, using this as a proxy for what is likely feeding shallow reservoirs of trans-crustal magma systems^33^. Our calculations are thus quantifying the range and variability of chemical compositions available for recharge that can contribute and impact the compositional diversity sampled in eruptions fed from shallow reservoirs. To test the impact of magma and heat extraction on the thermal and chemical evolution of magma reservoirs, we performed a subset of numerical simulations in which we extract 20, 40 and 60% of magma during the assembly of the magma reservoir.

**Figure 2:**
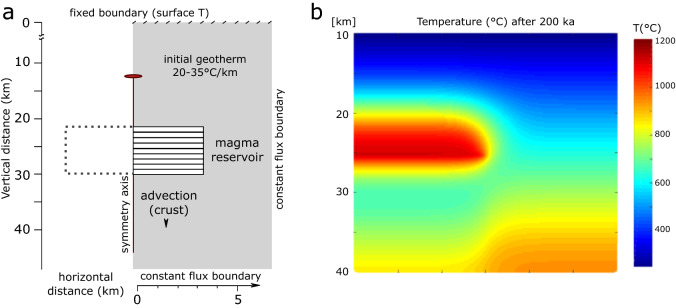
Numerical model to simulate the temporal evolution of temperatures in a crust undergoing pulsed magma injections. (**a**) Sketch illustrating the 2.5D axisymmetric modelling geometry. The left boundary of the model is an axis of rotational symmetry. The symmetry axis is indicated as red line on the left boundary of the model. Magma is instantaneously injected and under accreted as cylindrical sills, causing downward displacement of crustal rocks. The temporal evolution of the temperature field is calculated by solving the axisymmetric formulation of the heat conduction equation (“Methods” section). (**b**) Typical model output showing temperature (°C) variation in a 30 × 30 km crustal section after 200 ka of pulsed basaltic magma injection. Magma has accumulated with a centre at 24 km depth and core T > 1,100 °C. Like shown in (**a**) the left boundary of the model is modelled as a rotational symmetry axis.

In our model, basaltic magma is successively under-accreted as cylindrical, sill-shaped bodies starting at a crustal depth of 20 or 25 km, which causes downward displacement of the floor, and construction of magmatic reservoirs in a depth range between 20 and 30 km (Fig. 2, “Methods” section). We also tested different emplacement modes, but find in agreement with previous studies^22,34,35^ that the geometry of magma injection is of secondary importance for the thermal evolution of magmatic systems (c.f. “Methods” section). Previous thermal modelling studies have shown that in order to accumulate magma bodies in the Earth crust, reservoirs have to be fed by recharge pulses in close spatial and temporal association^22,23,34,35^. Such a scenario is consistent with rheological experiments and modelling, which show that the emplacement of a magma batch in the crust will attract further magma injection due to mechanical focussing of dykes and the rheological impediment exerted by the residing magma^36,37^. We thus simulate repetitive injection of magma over a similar depth range. To constrain the impact of modelling variables on the thermal evolution of incrementally-built^38,39^ magma reservoirs in the mid to lower crust, we varied the initial geothermal gradient (20–35 °C km^−1^) and vertical sill accretion rates from 0.002 to 0.0178 m year^−1^, corresponding to magma fluxes of between 0.0001 and 0.11 km^3^ year^−1^. The total duration of magma injection was between 100 and 800 ka, which is comparable to the timescales of typical composite volcanoes in arc settings, as constrained by geochronology^4–9,11,12^.

**Figure 3:**
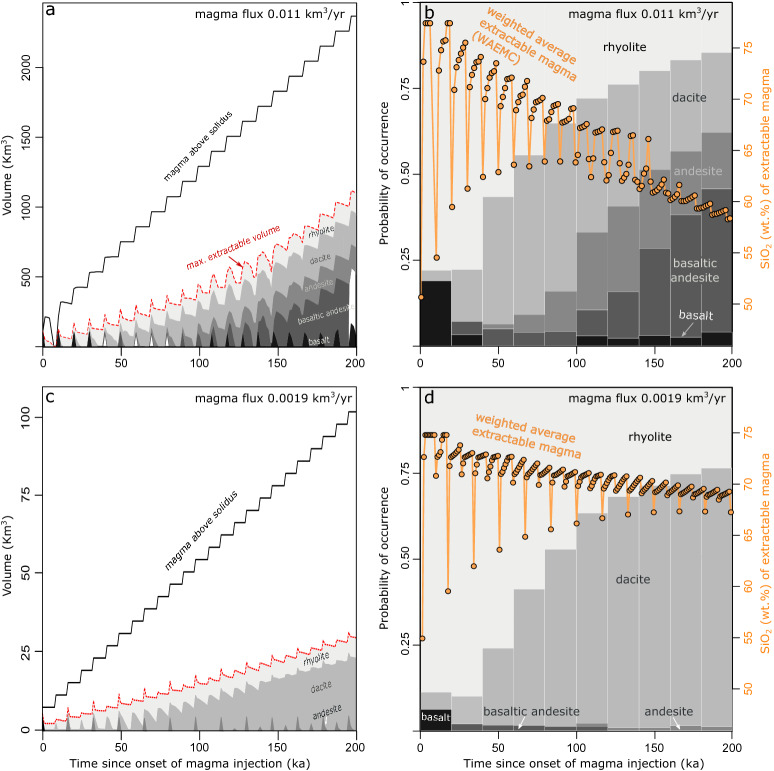
Coupling of thermal modelling output to experimental phase petrology. A geothermal gradient of 30 °C km^−1^ and sill injection depth of 25 km were used. (**a**) Temporal evolution of magma volumes above solidus (black curve) and maximum extractable magma volumes (red dashed curve) for a magma flux of 0.011 km^3^ year^−1^. The maximum extractable magma volume at each time since the onset of magma injection is calculated by adding magma volumes with less than 50 vol% crystallinity and all interstitial melt, using the melt fraction-temperature relation constrained from fractional crystallization experiments of Ref.^26^. The experimental relation of temperature and composition further permits to calculate the volumes of magma within compositional intervals (colour shading) that contribute to the maximum extractable volume, which is governed by the thermal structure of the reservoir. (**b**) Normalized volumes of mobile (i.e. potentially extractable) magma from (**a**) are shown with compositional colour shading as function of time since the onset of magma injection (left y-axis). Time evolution of the weighted average extractable magma composition (tan symbols, SiO_2_ wt%) is shown on the right y-axis to describe the central tendency and variance of mobile magma compositions. The system evolves towards more mafic compositions and less variability in time. (**c**) Time evolution of magma volumes as in (**a**) for a lower magma flux of 0.0019 km^3^ year^−1^. (**d**) Probability of occurrence and weighted average SiO_2_ (wt%) of extractable magma compositions as in (**b**) for lower magma flux.

We track the volumes of magma within different temperature intervals in the thermal model and assign a chemical composition and a crystallinity to the magma within each temperature interval according to the fractional crystallization experiments of Ref.^26^ (Table S2). Considering that magmas typically reach the rigid percolation threshold at about 50 vol% crystallinity^18,40–42^, we consider all magma below this threshold to be potentially extractable. Furthermore, we assume that interstitial liquids of magmas with more than 50 vol% crystals are potentially extractable by melt segregation or by mixing/mingling processes during disruption of highly crystallized crystal mushed associated with recharge^40–47^. Probabilities of melt extraction may significantly decrease in magma bodies with > 75% crystal content^41^. We thus tested the impact of reduced extraction probabilities of 2% for interstitial rhyolite melts from > 75% crystallised magma on our results. These calculations are presented in Fig. S5 and show equivalent trends at slightly more mafic conditions, when compared to simulations with no specified extraction probability. We therefore calculate the cumulative volume of extractable magma through time by adding at each time step the volume of magma with less than 50 vol% crystals and the volume of interstitial melt for magma with more than 50 vol% crystals (Fig. 3a, c).

We assume that the likelihood of a specific extractable magma composition to be sampled by a magma extraction event is only proportional to its volumetric fraction within the magma reservoir at extraction time. We do not assume any relationship between magma rheology and eruptibility nor do we consider a trigger mechanism. Hence, the probability (***P***) of a specific extractable magma composition to be extracted is given by:

1 $${\varvec{P}} = V_{ex}^{T} /V_{ex}^{tot}$$

where ${V}_{ex}^{T}$ is the volume of potentially extractable magma within a specified temperature range (i.e. specific chemistry) and ${V}_{ex}^{tot}$ is the total volume of extractable magma within the same time interval. Within this set of assumptions, we calculate the relative probability of different magma compositions to be extracted over time (Fig. 3b, d). Because in real systems magma might hybridize to various degrees by mixing or mingling processes^48,49^, we also calculate the weighted average extractable magma composition (WAEMC) over the simulated time period (orange line in Fig. 3b, d). Our calculations are thus quantifying the temporal evolution of magmas that experience fractional crystallization, melt segregation and hybridization processes.

## Results and discussion

### Temporal evolution of extractable magma chemistry

Figure 3b shows the probability of occurrence (***P***; Eq. 1) of chemical compositions in 20 ka steps over 200 ka for a simulation with magma flux of 0.011 km^3^ year^−1^ and intrusion depth of 25 km. Eruptions in the first 20 ka since the onset of magma injection in the crust have high probability of producing rhyolites, followed by basalt and minor dacite (or mixed compositions; WAEMC). The high probability of occurrence of chemically evolved magma during the early stages of evolution of a magma reservoir is driven by rapid magma cooling caused by the small system sizes and the steep temperature gradients between magma and colder crustal rocks. Such rapid cooling also causes intermediate magma composition to be present only for short timescales. With time, continued magma input into the system progressively heats up the crust. As a consequence, later during the evolution of a magmatic system, the probability of intermediate and mafic magmas to be extracted increases (Fig. 3b). Additionally, the variability of the WAEMC (Fig. 3b, d), drops progressively with time, showing that the thermal maturation of the system favours the extraction of progressively more chemically homogeneous magmas for systems that experience large-scale magma hybridization. Therefore, reservoirs assembled at the simulated conditions would initially produce recharge magmas of rather variable compositions (with WAEMC close to rhyolite), and progressively feed more mafic and less chemically variable reservoirs (Fig. 3b).

The results for a crustal magma reservoir built at 25 km depth by a volumetric magma flux of 0.0019 km^3^ year^−1^ over 200 ka are presented in Fig. 3d. As in the case of the system built by relatively high magma input (Fig. 3b), in the early stages of evolution the WAEMC is mainly rhyolitic with some minor basalts. While small volumes of intermediate and mafic magmas are present in the system at all times, the bulk of the reservoir is composed of rhyolite and dacite magma, the fraction of which increases with time. The WAEMC remains more felsic with respect to the previous case and evolves to only moderately more mafic chemistry with time (Fig. 3d). This represents a case, in which the chemistry of magma, feeding upper crustal reservoirs, would be fairly monotonous and silicic in time. Also in this case, the variance in extractable magma compositions and the general tendency towards less evolved compositions with time is well described by the WAEMC.

**Figure 4:**
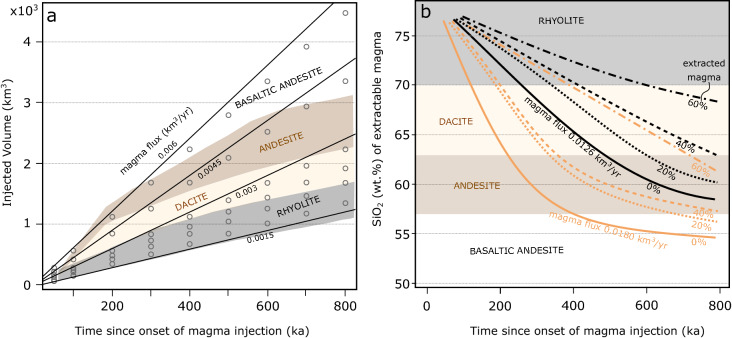
Temporal evolution of extractable magma chemistry. (**a**) The time since the onset of the magma injection episode (ka) is plotted versus the volume of injected magma (km^3^). Open symbols are individual thermal modelling runs and the solid lines were drawn for constant magma fluxes (km^3^ year^−1^). Colour shading represents the WAEMC of the built reservoirs. Numerical simulations were run with an initial geothermal gradient of 25 °C km^−1^ and for repeated injection of 100 m thick sills. The initial intrusion depth was set to 20 km. Note that for constant magma flux the extractable magma chemistry becomes more mafic with time. (**b**) Time since the onset of magma injection versus the SiO_2_ content of the extractable magma of the growing magma reservoirs. The solid black and orange lines are drawn for different constant magma fluxes of 0.0126 km^3^ year^−1^ and 0.0180 km^3^ year^−1^, respectively. Dashed lines indicate numerical simulations run under identical conditions, but with heat and mass extraction at a rate equivalent to 20, 40 or 60% of the base model (solid lines). Simulations with heat and mass extraction (dashed curves) lead to a shift towards more silicic compositions in time.

The results show that a constant rate of magma input into a mid-deep crustal reservoir tends to generate progressively less chemically evolved WAEMC with time (Fig. 4). For example, the composition of extractable magma from a reservoir assembled with a constant magma flux of 0.0045 km^3^ year^−1^ would be rhyolitic over the first 260 ka, become dacitic over the following 240 ka, and transition to andesitic about 500 ka after the onset of magma injection (Fig. 4a). Thus, a volcano erupting over 800 ka would sample a range of different magma compositions through time, which can be attributed to progressive magma addition and heating of the crust. At these injection rates, the reservoir is mostly comprised of crystal mush for prolonged times rather than eruptible melt, which emerges transiently in association with magma recharge. Higher magma injection rates (e.g. 0.006 km^3^ year^−1^), generate a different temporal evolution of WAEMC, with a transition (over ~ 300 ka) from silicic-dominated magmas to intermediate chemistries, followed by a prolonged phase dominated by monotonous basaltic andesites (Fig. 4a). The results indicate that volcanoes can erupt monotonous chemistry either in the later stages of their life or if the input of magma from the mantle decreases with time. Our modelling also indicates that compositionally contrasting monotonous volcanoes such as Nevado de Toluca (dacite; Fig. 1a) and Merapi (basaltic andesite; Fig. 1b) may be characterized by different magma fluxes. High magma flux regimes result in WAEMC that evolve towards mafic compositions over shorter timescales compared to cases with lower magma flux (Fig. 4).

In a subset of simulations, we tested the effect of magma (i.e. heat) extraction (e.g., due to volcanic eruptions) on the compositional evolution of WAEMC in crustal reservoirs. Generally, similar temporal trends towards more mafic compositions are observed, but for the same rate of magma input, magma extraction increases the time required for the WAEMC to become progressively more mafic. For the same rate of magma input, this timescale increases with the percentage of extracted magma (Fig. 4b). The results show that for extraction efficiencies of 20% and 40%, the difference in the 2σ SiO_2_ range of the compositional distributions with respect to calculations performed without extraction is always smaller than 4 wt% SiO_2_. Increasing the magma extraction efficiency to 60% results in differences in the 2σ SiO_2_ range of the WAEMC of < 5.5 wt% for intrusions built over 800 ka relative to simulations without extraction. These results indicate that the impact on compositional variability of heat and mass extraction is second order when the magmatic systems’ extraction efficiency is smaller than 20%.

**Figure 5:**
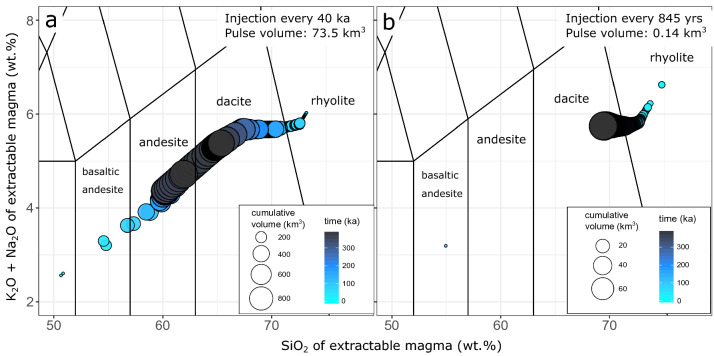
Impact of injection frequency on magma variability. Total alkali versus silica diagrams of the WAEMC for intrusions built with an identical vertical sill accretion rate of 0.006 m year^−1^ but different size and frequency of sill injections. Total duration of injection was set to 400 ka and an initial intrusion depth of 25 km. Symbol size is proportional to the volume of extractable magma, while the colour is proportional to time. (**a**) Injection of 73.5 km^3^ sills every 40 ka with an initial geothermal gradient of 30 °C km^−1^ and magma flux of 0.0018 km^3^ year^−1^ results in a broad compositional distribution. (**b**) Injection of 0.14 km^3^ in a single pulse every 845 years, initial geotherm of 35 °C km^−1^ and magma flux of 0.0001 km^3^ year^−1^ shows limited compositional diversity for the same vertical sill accretion rate of 0.006 m year^−1^ as in (**a**). Different geotherms were used in the presented simulations in order to present contrasting cases. Note that the differences in the cumulative volume of extractable magma between the two cases result from respecting the natural scaling between sill thickness and length^49^. Injection of sills with radii equal to the ones in (**a**) also produces less geochemical diversity compared to intrusion of thicker sills (Fig. S2).

Our calculations also show that for the same vertical sill accretion rate, both the size of magma pulses and the injection frequency affect the compositional diversity of extractable magmas (Fig. 5). Larger and more sporadic magma injection events (Fig. 5a) produce larger compositional diversity compared to frequent injection of small batches of magma (Fig. 5b). Such a difference is caused by the greater amplitude of heating and cooling cycles of intrusions built by larger and less frequent magma injections^22^. Hence, while a constant vertical accretion rate will produce a similar long-term evolution of the mean magma chemistry, the compositional variance in a specific period of volcanic activity increases with the size of the pulses. The spread in extractable magma compositions is also a function of the thermal state of the crust, where magma is injected. Magmatic reservoirs built in hotter crust or at deeper levels produce WAEMC of more homogenous chemistry with respect to systems assembled in shallower or colder crust, as they are thermally buffered by the temperature of the surrounding wall-rocks.

**Figure 6:**
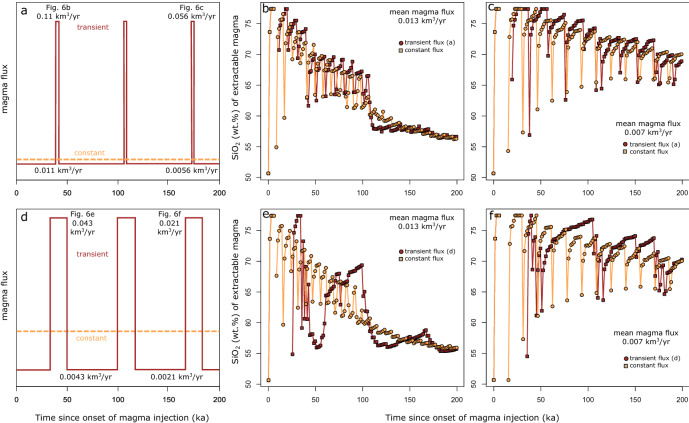
Impact of magma flux variation on trends and variance of average extractable magma chemistry. (**a**) Setup to simulate short-term, 1.7 ka lasting tenfold variation in magma flux (red line; arbitrary units) with time and equivalent constant magma flux (dashed tan line; arbitrary units). (**b**) Temporal evolution of average extractable magma chemistry (SiO_2_ wt%) for the scenario shown in (**a**). Transient magma flux variation is represented by red dots, the evolution for equivalent constant magma flux by tan squares. A mean magma flux of 0.013 km^3^ year^−1^ was used in this simulation. (**c**) Evolution of extractable magma chemistry, as in (**b**) with lower mean magma flux of 0.007 km^3^ year^−1^. (**d**) Scenario of long-term, 16.7 ka lasting tenfold magma flux variation (red line; arbitrary units) and equivalent constant recharge rate. (**e**) Impact of injection rate variation shown in (**d**) for a mean magma flux of 0.013 km^3^ year^−1^. (**f**) Compositional evolution of the extractable magma as in (**e**) for a lower average magma flux of 0.007 km^3^ year^−1^.

To explore the impact of variable magma flux^50^ on compositional trends and variability, we ran simulations in which the rate of magma input is increased periodically by a factor 10 for different time periods (Fig. 6). The results presented in Fig. 6a–c show that compositional variability and trends for simulations including short-lasting (e.g. 1.7 ka) increase of magma input are very similar to those without the transient increase of input and similar average rates of magma injection. This similarity remains even at higher temporal resolution than the most comprehensive geochronological studies of Arc volcanoes^4–8^. Long-lasting (e.g. 15 ka) episodes of increased magma injection rates (Fig. 6d, e) can result in differences in the chemistry of the extractable magma that are important enough to be potentially recognizable in natural datasets (Fig. 6e). This is particularly true for systems assembled by generally high magma fluxes.

### Compositional variability and recharge regimes

The compositional changes in the WAEMC calculated with our approach over 100 to 800 ka are comparable to geochronologically constrained eruptive histories of composite volcanoes in arc settings^6,7^ (Figs. 1, S3). Reconstruction of compositional trends for individual volcanoes requires detailed fieldwork in concert with dating and geochemical analysis, which to date is only available in a limited number of case studies^4–8,11,12^. Some of these studies find no obvious variations of chemistry with time^5^, however, trends towards more silicic compositions in time^51^, as well as the reverse sense of differentiation from silicic towards mafic^6, 7^ have been documented in the geological record. Although our calculations cannot capture the details of chemically resolved eruption records, they provide a framework to invert the chemistry of volcanic products (average erupted magma chemistry over time and chemical variability of eruptive products) and obtain quantitative information on fundamental parameters such as the long-term average rate of magma input in subvolcanic reservoirs.

As an example, the timescales of long-term chemical change from rhyodacitic towards basaltic andesite are about 60 ka for the well-characterized Parinacota volcano in the Central Andes^7^ (Fig. S3) and about 200 ka for the first half of the eruptive history of the Mazama-Crater Lake system in the Oregon Cascades^6^ (Fig. 1e). The direction and temporal relations of compositional change compare favourably with our modelling results in which magma reservoirs experience a temperature increase with time at a relatively constant injection rate. Our results show that extractable magma accumulating under relatively constant average rate of magma input in magma reservoirs invariably evolves towards more mafic and more homogeneous compositions in time. These general trends are also observed in model scenarios with reduced extraction probability of 2% for rhyolite compositions from highly crystallised (i.e. > 75%) magma (Fig. S5) and for simulations with removal of 20, 40 or 60% of the hottest magma. Thus, temporal trends toward more felsic erupted magma chemistry suggests a decrease of the average rate of magma input (Fig. 4). Recharge rates from the mantle may change over timescales of hundreds of thousands of years^52^, and a decrease of the rate of magma input or a pause in the recharge of the deep portion of the plumbing system could increase the average silica content of the volcanic products as observed for the recent history of Crater Lake and Puyehue-Cordón Caulle^8^ (Southern Volcanic Zone, Chile; Fig. 1).

**Figure 7:**
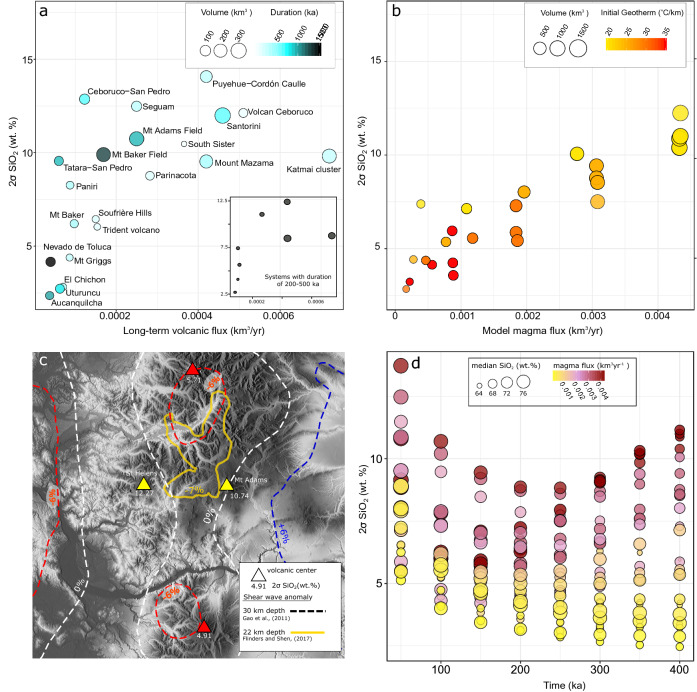
Relation of extractable magma diversity to injection rates and thermal conditions of the crust. (**a**) Long-term volcanic fluxes (Ref.^8^; Table S4) versus 2σ of extractable magma SiO_2_ content (wt%). Symbol size corresponds to the cumulative volume and colour contouring was done for the duration of magmatism. Inset shows relation for systems with duration between 200 and 500 ka only. (**b**) Relation of the 2σ range of extractable magma SiO_2_ content (wt%) and magma flux in model calculations. Symbol size corresponds to the cumulative volume and colour coding for initial geotherm. (**c**) Relief map of the central Cascades (USA) with and S-wave anomalies (dashed lines; Refs.^57,58^) compared to 2σ range of extractable magma SiO_2_ (wt%) for volcanic centres (triangles). Relief map data from OpenStreetMap contributors, CC BY-SA. (**d**) Cumulative 2σ range of extractable magma SiO_2_ content (wt%) versus time since onset of injection in ka. Symbol size is proportional to the median of extractable magma SiO_2_ (wt%) and colour coded for magma flux (km^3^ year^−1^).

The Age-SiO_2_ relations of Mount Mazama-Crater Lake^6^ and Puyehue-Cordón Caulle^8^ (Fig. 1e, f) also indicate that a large variety of compositions can be erupted in close temporal association at the later stages of volcano lifecycles. While the WAEMC converges towards more homogeneous compositions with time, an increasing variety of melt compositions may coexist in the later stages of a magmatic systems lifecycle (Fig. 3a, b), implying that a large variety of compositions may be feeding systems that lack large-scale magma mixing/mingling processes. Petrological data, which can either indicate a dominant role of magma hybridization^10,29^ or in other cases shows limited evidence for interaction of different melts^41,53^ may be used to evaluate, if erupted products of a particular eruptive centre are more consistent with a WAEMC or the diversity of coexisting melts. To compare our results with a larger set of volcanic systems, we complement the compilation of long-term volcanic fluxes from Ref.^8^ (Fig. 7a). We assume that time-averaged erupted volumes can be used as a proxy for the rate of magma input and the time integrated erupted volumes are proportional to the sizes of the magmatic plumbing systems. Estimates of time-averaged erupted volumes are affected by preservation issues and non-linearity in volumetric eruption rates. Variations of the volumetric eruption rate can be the result of changes in the rate of magma input^21^, but could also reflect higher eruption efficiency of already existing reservoirs due to crustal stress field changes resulting from mass redistribution processes (e.g. cone collapse or glacial unloading;^20,54–56^). Nevertheless, the compilation presented in Fig. 7a shows that larger volcanoes also have higher average volumetric output rates, which confirm that long-term eruption rates can serve as a proxy for crustal magma input. Importantly, long-term volcanic output rates are biased towards lower values with increasing age of the system^56^. Young systems with historic or Holocene records typically show higher eruptive fluxes compared to records built on long-term geological reconstructions. It is therefore critical to state that a comparison between compositional diversity and fluxes in the model and natural data is most robust for natural systems with a relatively long eruptive history.

The collected data show a clear tendency towards higher 2σ SiO_2_ ranges with increasing long-term volcanic flux (Fig. 7a), both when considering the full dataset and a subset of volcanoes filtered for similar duration of the volcanic activity (i.e. 200–500 ka). Furthermore, this analysis shows that larger volcanic systems like Santorini (~ 300 km^3^—11.99 wt% 2σ SiO_2_) are prone to erupt higher magma diversity compared to smaller size systems like El Chichón (2.82 wt% 2σ SiO_2_—26 km^3^). Our thermo-petrological model predicts both of these effects as reservoirs assembled at high rates of magma input accumulate WAEMC of a wide variety of compositions, which contrasts with the chemically homogeneous nature of extractable magmas in reservoirs assembled at low rates (Figs. 3a, b, 7b). Additionally, our calculations suggest that magmatic systems that are built in deeper and/or hotter crust develop less compositional diversity compared to more shallow crustal or lower geothermal gradient cases (Fig. 7b). Estimates of mid-deep crustal reservoir depths and geophysical data that could be used to constrain temperature or melt fraction are scarce. However, our results are consistent with the relationship between shear wave velocity anomalies and compositional diversity of volcanic systems in the central Cascades Ranges (USA; Fig. 7c). The compositionally monotonous centres Mt. Hood (4.91 wt% 2σ SiO_2_) and Mt Rainer (5.71 wt% 2σ SiO_2_) are underlain by a shear wave velocity decrease of 7% at a crustal depth of 30 km^57^. Mt. St. Helens (12.27 wt% 2σ SiO_2_) and Mt. Adams (10.74 wt% 2σ SiO_2_), at the high end of compositional diversity, show close proximity to a shallower seismic anomaly at 22 km depth^58^. On the other hand, the collected data highlight a lack of correlation between compositional variability and the lifespan of volcanic systems (Fig. 7a).

The comparison of our results with age resolved geochemical records of arc volcanoes indicates that such an approach has potential to be used to obtain first order estimates of the magma input and thermal conditions of the crust when applied to eruptive records of individual systems (Fig. 7d). Generally, volcanoes characterised by erupted magma with limited chemical variability such as Nevado de Toluca^12^, El Chichon^59^ and Uturuncu^11^ are associated with lower magma fluxes into relatively hot or deep crustal environments. Volcanoes erupting magmas with a wide range of compositions like Parinacota^7^, Mt Adams^60^, Mount St Helens^61^ best compare with models in which the rate of magma input is high and the subvolcanic reservoirs are assembled into relatively cold (or shallower) crust. Magma mixing, segregation and density filters have been all proposed to control the compositional variability of magmas sampled by volcanoes^19,41^. Our calculations show that the recharge rate and thermal conditions of the surrounding crust are further important variables to explain compositional variability at volcanoes. This does not rule out the potential contribution from other processes, but suggests that the thermal evolution of magma reservoirs plays a fundamental role in modulating the chemistry of volcanic products. Additionally, our results show that transition toward more mafic chemistry of volcanic products does not necessarily imply an increase in the average rate of magma input into the system. How thermally evolving crustal rheology^13–16^ modulates our results is discussed in the Supplementary information.

The thermochemical model presented here permits a quantitative connection to be established between variables such as magma flux, frequency of magma injection and thermal state of the crust surrounding magmatic systems and data commonly collected for volcanic systems such as age of the eruption and major element analyses (Fig. 7d)^4–12^. This approach creates opportunities to constrain extensive variables such as magma flux, which are crucial to reconstruct the mechanisms governing the chemical and physical evolution of magmas in the crust.

## Methods

We performed thermal modelling of pulsed magma injection into the mid to lower crust coupled with experimental phase relations^26^, which allows us to track the temporal evolution of extractable magma chemistry. Our model design builds on the theory that silicic magmas originate at mid to deep crustal levels^22–32^ and that magmatic bodies are built over protracted periods of time by incremental assembly^35,38,39^.

### Numerical modelling

We use the principles of heat conduction, derived by combining the conservation of energy and Fourier’s law, to describe the temporal and spatial evolution of temperature in a crust that experiences repeated magma injection. Our results were obtained by numerically solving the 2.5D (i.e., axisymmetric) formulation of the heat diffusion equation, which can be written as:

2 $$\rho c\frac{\partial T}{{\partial t}} = \frac{1}{r}\frac{\partial }{\partial r}\left( {rk\frac{\partial T}{{\partial r}}} \right) + \frac{\partial }{\partial z}\left( {k\frac{\partial T}{{\partial z}}} \right) + \rho L\frac{{\partial X_{c} }}{\partial t}$$

where *t* is the time, *T* is the temperature, *z* is the vertical coordinate, *r* is the radial distance from the axis of rotational symmetry, *k* is the thermal conductivity, *L* is the latent heat of crystallization, *ρ* is the density, *c* is the specific heat and *X*_*c*_ is the fraction of crystals. Equation (2) was discretized and solved using an explicit finite difference method. Latent heating, due to the crystallization of magma, was implemented using the relation of temperature (*T*) and melt fraction (1 − *X*_*c*_), as derived from fractional crystallization experiments of a hydrous arc type basalt to rhyolite^26^. As *X*_*c*_ and *T* are nonlinearly dependent on each other, the governing equation was solved iteratively. The dependence of thermal conductivity (*k*) on temperature was implemented using the relations presented in Ref.^62^ for average crust. In all simulations, zero heat flux was imposed in the perpendicular direction to all lateral boundaries of the modelled space, apart from the surface, where temperature was fixed at 8 °C. In order to test the reliability of our results, we benchmarked the model against an existing thermal code^63,64^ that was solved with a different numerical method.

We model magma injection by modifying the temperature field at an initial crustal intrusion depth of 20 or 25 km, which is equivalent to increasing the initial geothermal gradient, and successive under-accretion of new basaltic sills at their liquidus temperature of 1,170 °C. In this setup, space is generated for freshly injected magma by downward advection of the host rocks. We also tested models with different emplacement geometry, in which magma was injected into the centre of the intrusion. Results obtained in this way differ from cases where under accretion is the emplacement style by higher average temperatures (Supplementary figure S1). Maximum temperature differences of about 25 °C between the different modelling geometries for a magma injection episode of 400 ka translate into 3 wt% SiO_2_ difference for a case where the intrusion is at a temperature corresponding to the maximum difference in the SiO_2_-*T* relation. This, however, does not alter the general conclusions drawn in this study.

To ensure comparability between different numerical runs, all presented results were derived from intrusions that were built by under accretion. We assumed a linear initial geothermal gradient, which was varied between 20 and 35 °C km^−1^ for individual numerical simulations. The duration of magma injection was systematically changed between 100 and 800 ka. To test the effect of different sill dimensions, we varied the thickness and radii of injected sills between 5 to 234 m and 3 to 20 km, respectively. As sill thickness and length are not independent of each other^65^, we respected the natural scaling between these parameters in most simulations. However, we also tested the influence of decreasing thickness at constant sill radius of 10 km. For a list of modelling variables, the reader is referred to Supplementary Table S1.

To test the impact of heat and mass removal on the thermal and compositional evolution during magma body construction, we implemented the possibility of magma extraction in the model. In a subset of simulations, magma was removed at a rate that is equivalent to 20, 40 or 60% of the final intrusion volume built by a particular magma flux, by imposing an appropriate extraction rate. Extraction sites were customized to match the previous injection site and dimension, which ensures removal of magma at the highest temperature in the reservoir, by advecting the temperature field upwards. This scenario was chosen under the assumption that the most mobile magma in the reservoir is represented by the highest temperatures.

### Petrologic calculations

To quantify the temporal evolution of extractable magma chemistry in the built intrusions we performed petrological modelling. Magmas are able to flow until they reach their rheological locking point, typically at about 50 vol% crystals^18^, while interstitial melt is considered to be able to leave the rigid crystal networks beyond the locking point by various segregation processes. Thus, we defined the extractable magma compositions as magma with < 50 vol% crystals and all interstitial melt. To calculate this composition, we tracked magma volumes within temperature intervals of 50 °C between the solidus *T*_*s*_ at 650 °C and 1,150 °C (intervals 1 to 9), with an additional 20 °C increment between 1,150 °C and the liquidus temperature *T*_*L*_ of 1,170 °C (interval 10) in the numerical model as functions of time. For each temperature interval we assigned average melt and bulk magma major element oxide compositions (wt%) and average melt fraction using the petrological experiments of Ref.^26^ (Supplementary table S2). These experiments were chosen because they represent the complete fractional crystallization sequence of a typical hydrous arc basalt to rhyolite at mid to lower crustal levels (0.7 GPa) that compares well to other experimental datasets in terms of evolutionary trends in major element components. Implementing a different temperature-chemistry relation in the model would shift the calculated values in compositional space depending on the particular relation used, but leads to more mafic chemistries and more homogeneous WAEMC with time (Fig. S6), which is equivalent to our findings and does therefore not alter the conclusions drawn in this study. Magma evolution in our model does not account for crustal melting, which has a typical productivity of < 10% for most lithologies at the crustal depth considered here^66^.

We calculated the mass of melt m_m_ and crystals m_x_ in each temperature interval along the temporal sequence by:

3 $$m_{m}^{i} = X_{m} { }V_{m } \rho_{m}$$

4 $$m_{x}^{i} = X_{c} { }V_{m } \rho_{x} ,$$

where ${X}_{m}$ and ${X}_{c}$ are the volumetric fractions of melt and crystals, *V*_*m*_ is the volume of magma in temperature interval *i*, ${\rho }_{m}$ and ${\rho }_{x}$ are the densities of melt and crystals, which were assumed to be 2,800 kg m^−3^ for the liquid and 3,300 kg m^−3^ for crystals. The total mass of extractable magma *M*_*e*_ (kg) at each time step is calculated by summation over all temperature intervals that contain interstitial melt (i = 1–10) and the temperature intervals corresponding to magma with less than 50% crystallinity (i = 7–10):

5 $$M_{e} = \mathop \sum \limits_{i = 1}^{10} m_{m}^{i} + \mathop \sum \limits_{i = 7}^{10} m_{x}^{i}$$

The chemical composition of extractable magma (${C}_{extract}$) in terms of the composition of its major element components (*C*_*x*_) is calculated as:

6 $$C_{extract} = \mathop \sum \limits_{i = 1}^{6} \frac{{m_{m}^{i} C_{X}^{i} }}{100} + \mathop \sum \limits_{i = 7}^{10} \frac{{\left( {m_{m}^{i} + m_{x}^{i} } \right)}}{100}C_{X}^{liq} ,$$

where ${C}_{X}^{liq}$ is the composition of the specific major element component at the liquidus temperature and *i* specifies the index for each temperature interval. Finally, the weighted average extractable magma composition (WAEMC) of the built reservoir with time in weight percent $({C}_{wt}$) for each major element component is constrained as:

7 $$C_{wt} = 100 \frac{{C_{extract} }}{{M_{e} }}$$

### Data compilation

In order to compare our modelling results to natural observations we compiled geochemical and age data for various volcanic systems in arc settings for which detailed studies of their eruptive histories are available (e.g. Parinacota^7^, Mazama^6^, El Chichón^59^, Nevado de Toluca^12^, St. Helens^61^, Puyehue Cordon Caulle^8^, Uturuncu^11^, Supplementary table 4). Care was taken to exclude prominent eruptions that have been sampled and analysed much more frequently, such as Mount St. Helens 1980 and the Upper Toluca Pumice. Geochronological studies have shown that eruption rates can vary dramatically throughout the lifespan of volcanoes with long periods of limited or no activity between eruptive cycles^4^. To make our continuous injection model comparable to long lived systems that show long lasting lulls in activity (Uturuncu, Nevado de Toluca), we compiled data that represents cone-building stages of these systems. Data for Merapi and Puyehue Cordón Caulle presented in Fig. 1 was mined from the GEOROC database (https://georoc.mpch-mainz.gwdg.de/georoc/) and new major element whole rock analyses are provided for Nevado de Toluca.

### Whole rock analysis

To extend the database we present geochemical analysis of 40 new bulk-rock samples from Nevado de Toluca volcano in Central Mexico. The analysed units’ span the entire eruptive history of the volcano and are either stratigraphically constrained or have been dated previously by radiocarbon or ^40^Ar/^39^Ar geochronology^12^. All samples were cleaned, washed and soaked in de-ionized water over night before they were dried in an oven at 50 °C. The dried rocks were then crushed and reduced to powders using an agate mill. The powdered material was mixed with Li-tetraborate and fused at 950 °C. Analyses for major elements were carried out at the X-ray fluorescence (XRF) laboratory at the University of Lausanne using a PANALYTICAL Axios^max^. The composition of several international reference materials (SY-2, TS-2, BHVO, NIM-N, NIM-G, BE-N) has been determined before and after sample analyses in the same analytical session.

## Supplementary information

Supplementary file1.

## Data Availability

Data that supports the findings of this study are available within the paper and supplementary information.

## Code availability

The numerical code that was used in this study is available from the corresponding author upon request.

## References

[1] Hedenquist JW, Lowenstern JB (1994). The role of magmas in the formation of hydrothermal ore deposits. Nature.

[2] Caricchi L, Blundy J (2015). The temporal evolution of chemical and physical properties of magmatic systems. Geol. Soc. Spec. Publ..

[3] Huber C, Bachmann O, Manga M (2009). Homogenization processes in silicic magma chambers by stirring and mushification (latent heat buffering). Earth Planet. Sci. Lett..

[4] Hildreth W (2007). Quaternary Magmatism in the Cascades: Geologic perspectives.

[5] Frey HM, Lange RA, Hall CM, Delgado-Granados H (2004). Magma eruption rates constrained by 40Ar/39Ar chronology and GIS for the Ceboruco-San Pedro volcanic field, western Mexico. Geol. Soc. Am. Bull..

[6] Bacon CR, Lanphere MA (2006). Eruptive history and geochronology of Mount Mazama and the Crater Lake region, Oregon. Geol. Soc. Am. Bull..

[7] Hora JM, Singer BS, Wörner G (2007). Eruptive flux during periods of cone growth and collapse at Volcan Parinacota, Chilean CVZ, from a high-resolution ^40^Ar/^39^Ar eruptive chronology. Geol. Soc. Am. Bull..

[8] Singer BS, Jicha BR, Harper MA, Naranjo JA, Lara LE, Moreno-Roa H (2008). Eruptive history, geochronology, and magmatic evolution of the Puyehue-Cordón Caulle volcanic complex, Chile. Geol. Soc. Am. Bull..

[9] Gertisser R, Keller J (2003). Temporal variations in magma composition at Merapi Volcano (Central Java, Indonesia): magmatic cycles during the past 2000 years of explosive activity. J. Volcanol. Geotherm. Res..

[10] Kent AJ, Darr C, Koleszar AM, Salisbury MJ, Cooper KM (2010). Preferential eruption of andesitic magmas through recharge filtering. Nat. Geosci..

[11] Muir DD, Barfod DN, Blundy JD, Rust AC, Sparks RSJ, Clarke KM (2015). The temporal record of magmatism at Cerro Uturuncu, Bolivian Altiplano. Geol. Soc. Spec. Publ..

[12] Torres-Orozco R, Arce JL, Layer PW, Benowitz JA (2017). The Quaternary history of effusive volcanism of the Nevado de Toluca area, Central Mexico. J. S. Am. Earth Sci..

[13] Jellinek AM, DePaolo DJ (2003). A model for the origin of large silicic magma chambers: precursors of caldera-forming eruptions. Bull. Volcanol..

[14] Gregg PM, De Silva SL, Grosfils EB (2013). Thermomechanics of shallow magma chamber pressurization: implications for the assessment of ground deformation data at active volcanoes. Earth Planet. Sci. Lett..

[15] Degruyter W, Huber C (2014). A model for eruption frequency of upper crustal silicic magma chambers. Earth Planet. Sci. Lett..

[16] Karlstrom L, Paterson SR, Jellinek AM (2017). A reverse energy cascade for crustal magma transport. Nat. Geosci..

[17] Stolper E, Walker D (1980). Melt density and the average composition of basalt. Contrib. Mineral. Petrol..

[18] Marsh BD (1981). On the crystallinity, probability of occurrence, and rheology of lava and magma. Contrib. Mineral. Petrol..

[19] Kent AJ (2013). Preferential eruption of andesitic magmas: implications for volcanic magma fluxes at convergent margins. Geol. Soc. Spec. Publ..

[20] Pinel V, Jaupart C (2000). The effect of edifice load on magma ascent beneath a volcano. Philos. Trans. R. Soc. A.

[21] Wörner G, Mamani M, Blum-Oeste M (2018). Magmatism in the Central Andes. Elements.

[22] Annen C, Blundy JD, Sparks RSJ (2005). The genesis of intermediate and silicic magmas in deep crustal hot zones. J. Petrol..

[23] Dufek J, Bergantz GW (2005). Lower crustal magma genesis and preservation: a stochastic framework for the evaluation of basalt–crust interaction. J. Petrol..

[24] Solano JMS, Jackson MD, Sparks RSJ, Blundy JD, Annen C (2012). Melt segregation in deep crustal hot zones: a mechanism for chemical differentiation, crustal assimilation and the formation of evolved magmas. J. Petrol..

[25] Jackson MD, Blundy J, Sparks RSJ (2018). Chemical differentiation, cold storage and remobilization of magma in the Earth’s crust. Nature.

[26] Nandedkar RH, Ulmer P, Müntener O (2014). Fractional crystallization of primitive, hydrous arc magmas: an experimental study at 0.7 GPa. Contrib. Mineral. Petrol..

[27] Müntener O, Ulmer P (2018). Arc crust formation and differentiation constrained by experimental petrology. Am. J. Sci..

[28] Laumonier M, Scaillet B, Pichavant M, Champallier R, Andujar J, Arbaret L (2014). On the conditions of magma mixing and its bearing on andesite production in the crust. Nat. Commun..

[29] Weber G, Arce JL, Ulianov A, Caricchi L (2019). A recurrent magmatic pattern on observable timescales prior to Plinian eruptions from Nevado de Toluca (Mexico). J. Geophys. Res. Solid Earth.

[30] Hill GJ, Caldwell TG, Heise W, Chertkoff DG, Bibby HM, Burgess MK, Cull JP, Cas RA (2009). Distribution of melt beneath Mount St Helens and Mount Adams inferred from magnetotelluric data. Nat. Geosci..

[31] Heise W, Caldwell TG, Bibby HM, Bennie SL (2010). Three-dimensional electrical resistivity image of magma beneath an active continental rift, Taupo Volcanic Zone, New Zealand. Geophys. Res. Lett..

[32] Comeau MJ, Unsworth MJ, Ticona F, Sunagua M (2015). Magnetotelluric images of magma distribution beneath Volcán Uturuncu, Bolivia: implications for magma dynamics. Geology.

[33] Cashman KV, Sparks RSJ, Blundy JD (2017). Vertically extensive and unstable magmatic systems: a unified view of igneous processes. Science.

[34] Annen C, Pichavant M, Bachmann O, Burgisser A (2008). Conditions for the growth of a long-lived shallow crustal magma chamber below Mount Pelee volcano (Martinique, Lesser Antilles Arc). J. Geophys. Res. Solid Earth.

[35] Karakas O, Degruyter W, Bachmann O, Dufek J (2017). Lifetime and size of shallow magma bodies controlled by crustal-scale magmatism. Nat. Geosci..

[36] Karlstrom L, Dufek J, Manga M (2010). Magma chamber stability in arc and continental crust. J. Volcanol. Geotherm. Res..

[37] Galetto F, Acocella V, Caricchi L (2017). Caldera resurgence driven by magma viscosity contrasts. Nat. Commun..

[38] Coleman DS, Gray W, Glazner AF (2004). Rethinking the emplacement and evolution of zoned plutons: geochronologic evidence for incremental assembly of the Tuolumne Intrusive Suite. Calif. Geol..

[39] de Saint Blanquat M, Horsman E, Habert G, Morgan S, Vanderhaeghe O, Law R, Tikoff B (2011). Multiscale magmatic cyclicity, duration of pluton construction, and the paradoxical relationship between tectonism and plutonism in continental arcs. Tectonophysics.

[40] Bachmann O, Bergantz GW (2004). On the origin of crystal-poor rhyolites: extracted from batholithic crystal mushes. J. Petrol..

[41] Dufek J, Bachmann O (2010). Quantum magmatism: magmatic compositional gaps generated by melt-crystal dynamics. Geology.

[42] Vigneresse JL, Barbey P, Cuney M (1996). Rheological transitions during partial melting and crystallization with application to felsic magma segregation and transfer. J. Petrol..

[43] Sisson TW, Bacon CR (1999). Gas-driven filter pressing in magmas. Geology.

[44] Hartung E, Weber G, Caricchi L (2019). The role of H_2_O on the extraction of melt from crystallising magmas. Earth Planet. Sci. Lett..

[45] Pistone M, Caricchi L, Ulmer P, Reusser E, Ardia P (2013). P Rheology of volatile-bearing crystal mushes: mobilization vs. viscous death. Chem. Geol..

[46] Huber C, Parmigiani A (2018). A physical model for three-phase compaction in silicic magma reservoirs. J. Geophys. Res. Solid Earth.

[47] Floess D, Caricchi L, Simpson G, Wallis SR (2019). Melt segregation and the architecture of magmatic reservoirs: insights from the Muroto sill (Japan). Contrib. Mineral. Petrol..

[48] Maughan LL, Christiansen EH, Best MG, Gromme CS, Deino AL, Tingey DG (2002). The Oligocene Lund Tuff, Great Basin, USA: a very large volume monotonous intermediate. J. Volcanol. Geotherm. Res..

[49] Hildreth W, Wilson CJ (2007). Compositional zoning of the Bishop Tuff. J. Petrol..

[50] Schöpa A, Annen C (2013). The effects of magma flux variations on the formation and lifetime of large silicic magma chambers. J. Geophys. Res. Solid Earth.

[51] Turner S, Caulfield J, Rushmer T, Turner M, Cronin S, Smith I, Handley H (2012). Magma evolution in the primitive, intra-oceanic Tonga arc: rapid petrogenesis of dacites at Fonualei volcano. J. Petrol..

[52] de Silva SL, Kay SM (2018). Turning up the heat: high-flux magmatism in the Central Andes. Elements.

[53] Weber G, Castro JM (2017). Phase petrology reveals shallow magma storage prior to large explosive silicic eruptions at Hekla volcano, Iceland. Earth Planet. Sci. Lett..

[54] Jicha BR, Laabs BJ, Hora JM, Singer BS, Caffee MW (2015). Early Holocene collapse of Volcán Parinacota, central Andes, Chile: Volcanological and paleohydrological consequences. Bulletin.

[55] Maclennan J, Jull M, McKenzie D, Slater L, Grönvold K (2002). The link between volcanism and deglaciation in Iceland. Geochem. Geophys..

[56] Watt SF (2019). The evolution of volcanic systems following sector collapse. J. Volcanol. Geotherm. Res..

[57] Gao H, Humphreys ED, Yao H, van der Hilst RD (2011). Crust and lithosphere structure of the northwestern US with ambient noise tomography: Terrane accretion and Cascade arc development. Earth Planet. Sci. Lett..

[58] Flinders AF, Shen Y (2017). Seismic evidence for a possible deep crustal hot zone beneath Southwest Washington. Sci. Rep..

[59] Layer PW, García-Palomo A, Jones D, Macías JL, Arce JL, El Mora JC (2009). Chichón volcanic complex, Chiapas, México: stages of evolution based on field mapping and ^40^Ar/^39^Ar geochronology. Geofís. Int..

[60] Hildreth W, Lanphere MA (1994). Potassium-argon geochronology of a basalt-andesite-dacite arc system: The Mount Adams volcanic field, Cascade Range of southern Washington. Geol. Soc. Am. Bull..

[61] Clynne MA, Calvert AT, Wolfe EW, Evarts RC, Fleck RJ, Lanphere MA (2008). he Pleistocene eruptive history of Mount St. Helens, Washington, from 300,000 to 12,800 years before present: Chapter 28. A Volcano Rekindled: The Renewed Eruption of Mount St. Helens, 2004–2006.

[62] Whittington AG, Hofmeister AM, Nabelek PI (2009). Temperature-dependent thermal diffusivity of the Earth’s crust and implications for magmatism. Nature.

[63] Caricchi L, Simpson G, Schaltegger U (2014). Zircons reveal magma fluxes in the Earth’s crust. Nature.

[64] Caricchi L, Simpson G, Schaltegger U (2016). Estimates of volume and magma input in crustal magmatic systems from zircon geochronology: the effect of modeling assumptions and system variables. Front. Earth Sci..

[65] Cruden AR, Kenneth JWM, Bunger AP (2017). Geometric scaling of tabular igneous intrusions implications for emplacement and growth. Physical Geology of Shallow Magmatic Systems.

[66] Dufek J, Huber C, Karlstrom L (2013). Magma chamber dynamics and thermodynamics. Modeling Volcanic Processes.

