# Supplementary material for: Magma diversity reflects recharge regime and thermal structure of the crust
Source: Sci Rep. 2020 Jul 17;10:11867. doi: 10.1038/s41598-020-68610-1 (PMC7368054; doi:10.1038/s41598-020-68610-1)
Supplement: Supplementary file 1 — Supplementary file1. [file 41598_2020_68610_MOESM1_ESM.pdf]

Supplementary information

Magma diversity reflects recharge regime and thermal structure of the crust

Gregor Weber*, Guy Simpson, Luca Caricchi

Department of Earth sciences, University of Geneva, 1205 Geneva, Switzerland

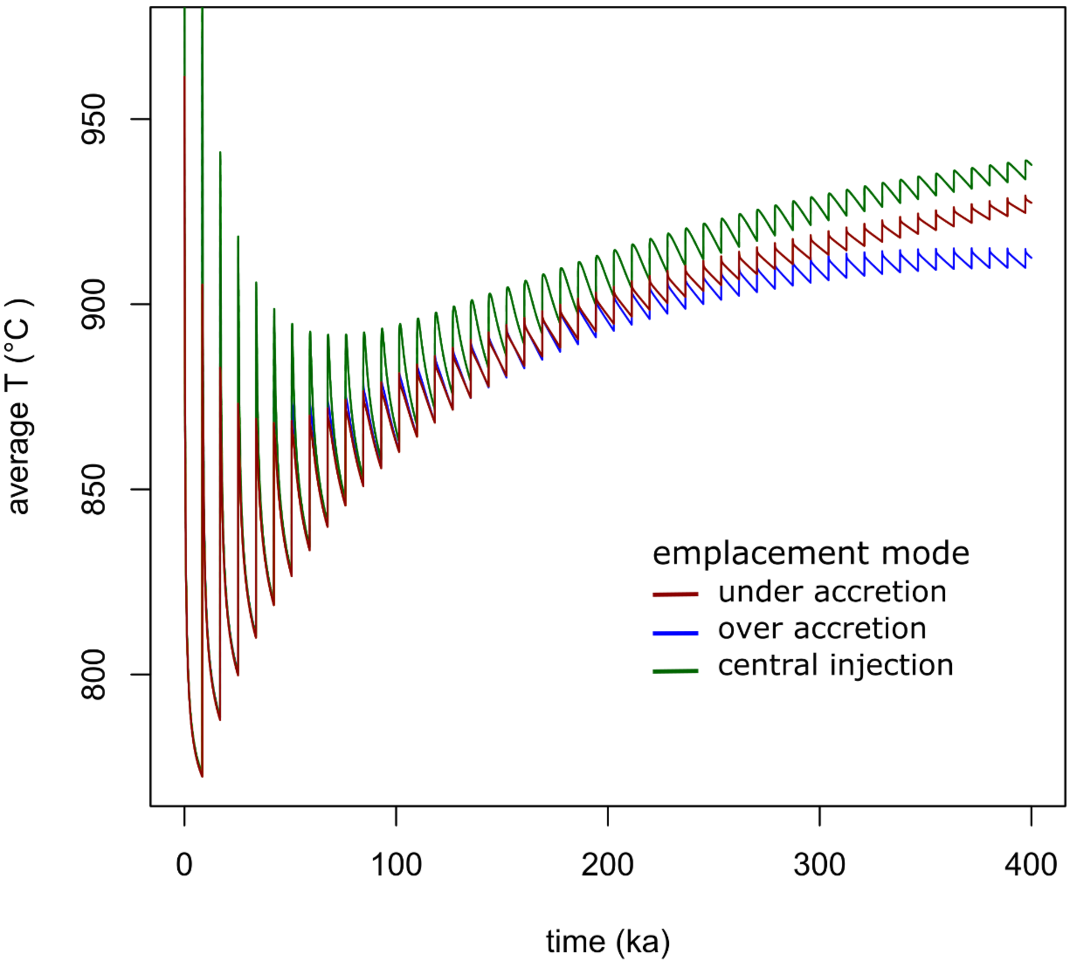

Figure S1: Impact of emplacement mode on average temperature of the intrusion. Numerical simulations were run with identical parameters except for the geometry of emplacement. Initial geothermal gradient of 30°C/km, vertical accretion rate of 0.0059 m/yr and initial injection depth of 25 km were used. Intrusions built by over accretion have lower average temperatures compared to magma reservoirs emplaced by under accretion of injection into the central part. An increase of average temperature with time is observed for all tested emplacement modes.

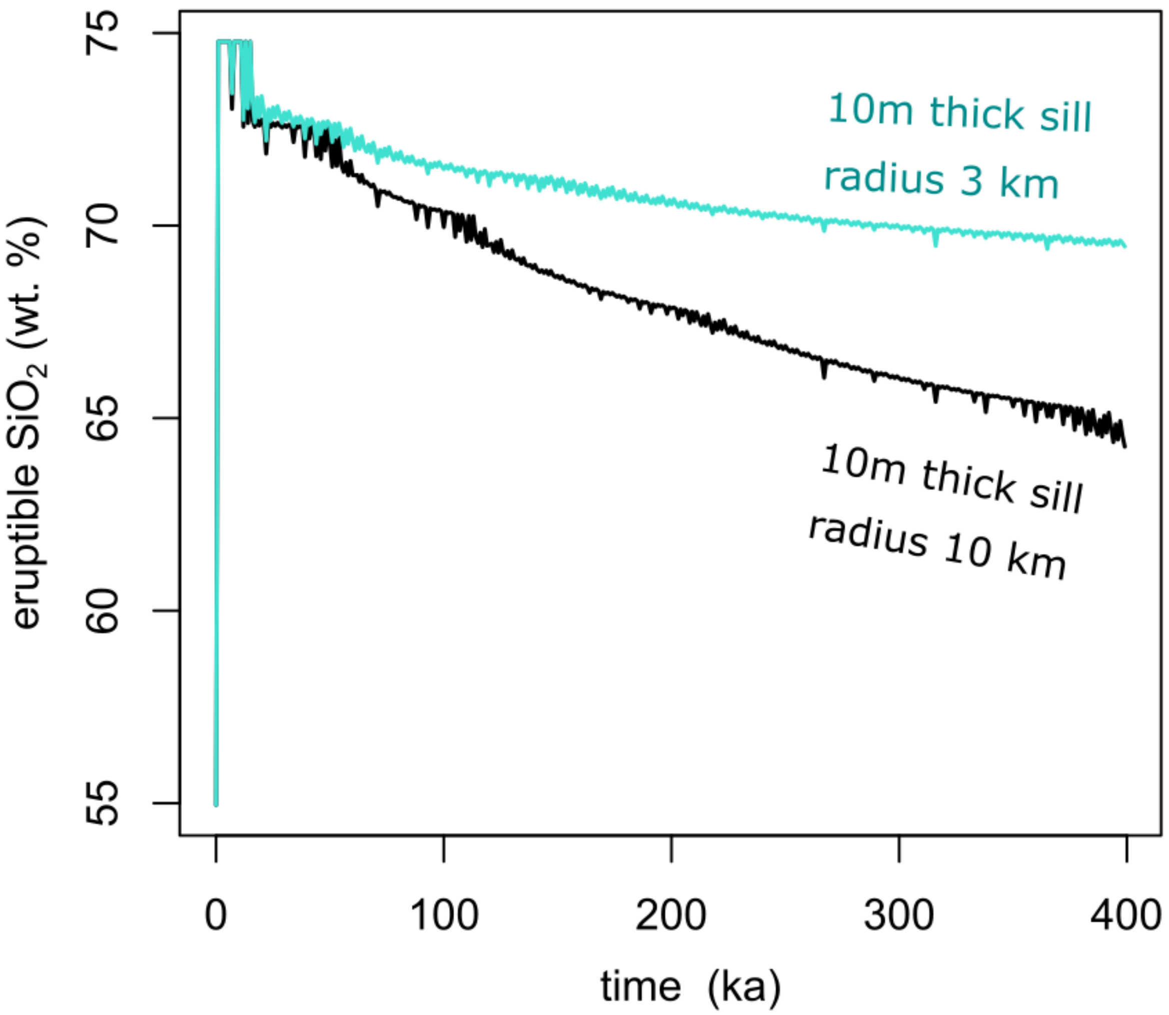

Figure S2: Effect of sill dimensions on thermal evolution of incrementally assembled intrusions. Injection of 10m thick sills with radius of 10 km (black) and 3 km (turquoise) into the middle crust with initial geothermal gradient of 30°C/km. Note that injection of small batches results in less chemical diversity with time.

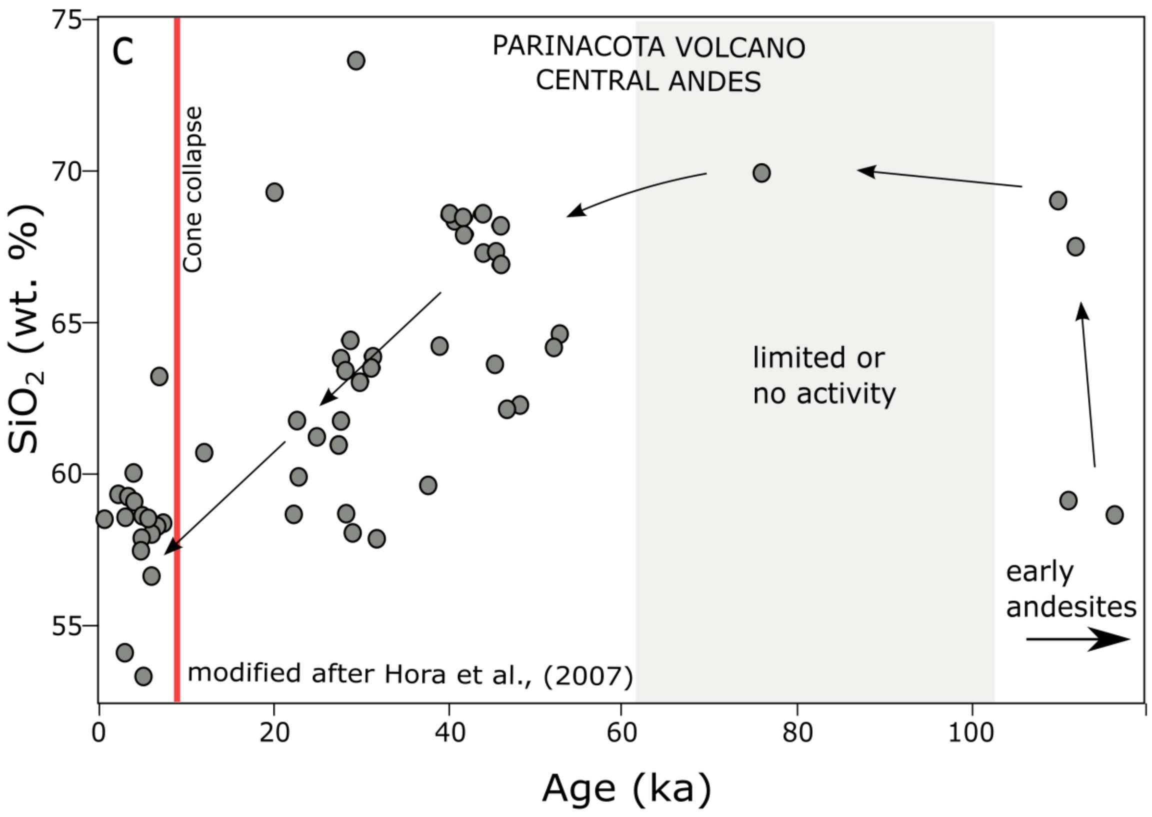

Figure S3: Eruptive history of the Parinacota volcano in the Central Andean Volcanic Zone, modified from Hora et al., (2007). SiO_2 (wt. %) whole rock contents become more mafic in time for the younger history of this volcano as indicated by arrows. The volcano has collapsed and produced as 6 km^3 debris avalanche at about 8.8 ka (Jicha et al., 2015). The most mafic compositions and highest volumetric eruption rates are observed after the collapse event.

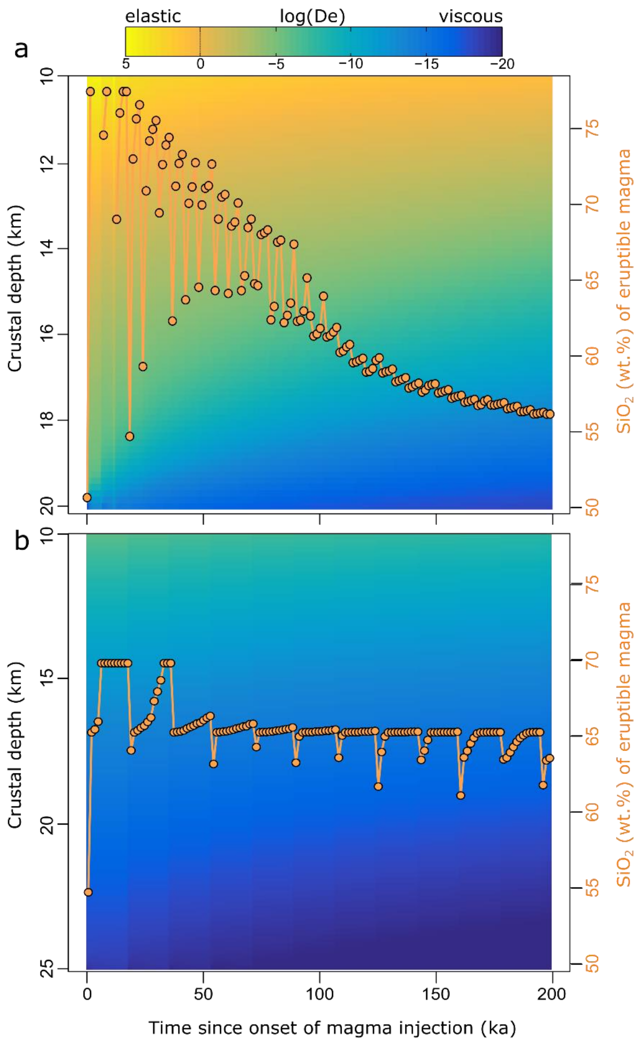

Figure S4: Time evolution of crustal Deborah Number (De) and mobile magma chemistry. The time since the onset of magma injection (ka) is plotted versus crustal depth overlying the magma reservoir (left y-axis), measured from the top of the intrusion centre. Colour shading specifies the temporal evolution of De at various crustal depth. De>>1 indicates an elastically dominated crustal response, while De<<1 indicates viscous behaviour of crustal rocks. The weighted average SiO_2 (wt. %) of the eruptible magma for the underlying intrusion through time is shown on the right y-axis. Two endmember scenarios are shown. a) High magma flux (0.0178 km^3/yr) case with relatively low geothermal gradient (20 °C/km). b) Low magma flux of 0.00056 km^3/yr and geotherm of 35 °C/km. Note that the elastic or viscous response of crustal rocks (De) is highly dependent on the temperature of the crust. Changing the initial geothermal gradient to lower values will therefore shift De to more elastic rheologies, while low magma fluxes still produce less chemical variability compared to high magma flux cases.

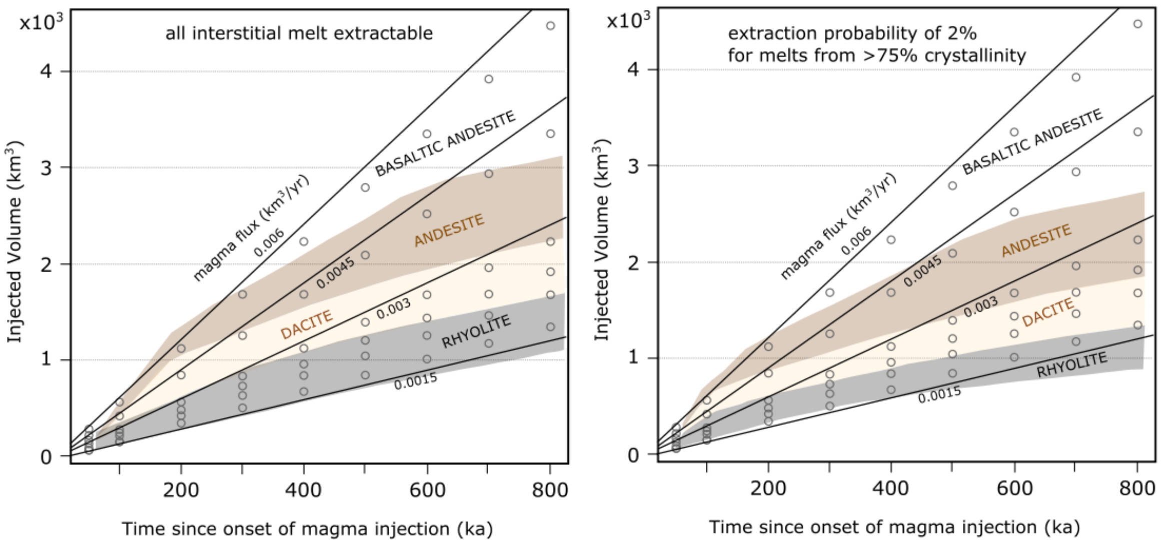

Figure S5: Comparison of simulations with different probabilities of melt extraction. Time since the onset of magma injection is plotted versus injected volume. Solid black lines were drawn for different constant magma fluxes. Open symbols reflect individual numerical simulations. Colour shading represents WAEMC. Left: All interstitial melt considered extractable. Right: Reduced probability of 2% for rhyolite melt extraction from >75% crystallized magma reservoirs.

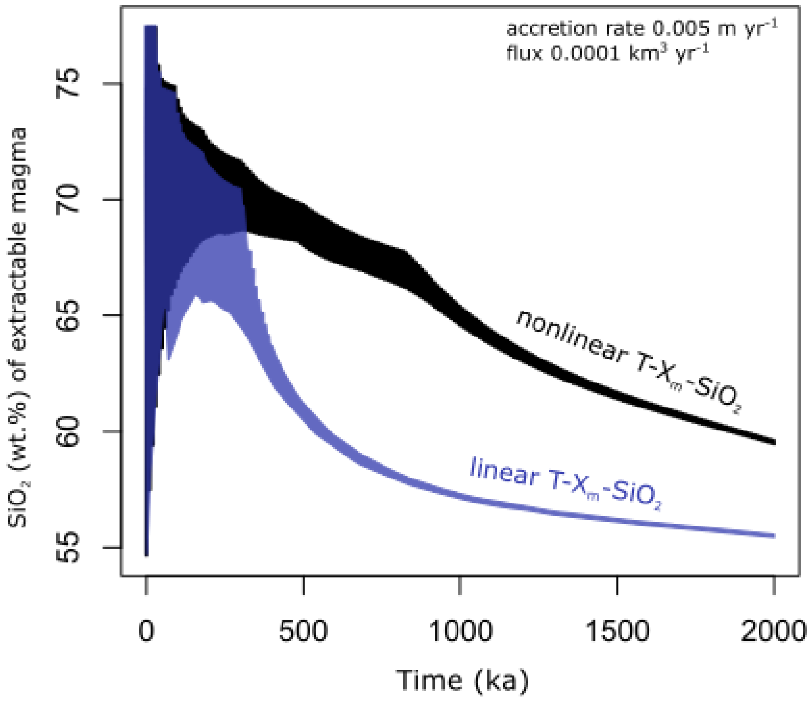

Figure S6: Time evolution of the SiO2 (wt.%) of the extractable magma (WAEMC) for different melt-fraction, temperature, composition relations. The black curve was modelled using the non-linear experimental relation of Nandedkar et al. (2014), while the blue curve was calculated using a fully hypothetical linear relation of melt-fraction, temperature and SiO_2. Note that both curves evolve towards more mafic compositions in time and show less variability with time.

Supplementary discussion: Crustal rheology

To compare how the compositional evolution of magmatic systems compares to magma transfer and eruption mechanics, we use the thermal modelling results to calculate the Deborah Number (De), which quantifies the mechanical response of crustal wall rocks subjected to magma injection. Following the approach presented in Karlstrom et al., (2017), De is proportional to the ratio of a magma injection related inverse strain rate ($t_{inject} \sim V_{res}/Q_{in}$) and the Maxwell viscoelastic relaxation timescale ($t_{relax} \sim \mu/G$):

$$De \sim \frac{(\mu\, Q_{in})}{(V_{res}\, G)} \qquad (S1),$$

where μ is the viscosity of crustal rocks, Q_{in} is the average volumetric magma input rate, V_{res} is the volume of the magma reservoir through time, and G is an effective elastic shear modulus, which was assumed to be 10 GPa. The volumetric magma flux (Q_{in}) and volume of the magma reservoir through time V_{res} were tracked in the thermal model. We calculated the crustal viscosity (μ) as a function of crustal depth and time, under the assumption of granitic crust, using the temperature distribution of rocks overlying the intrusion centre and an Arrhenius law of the form:

$$\mu = A_{\mu}\, e^{\frac{A}{(R\,T)}} \qquad (S2),$$

where A_{μ} is an empirical constant of 4.25×10^7, A is the activation energy 131×10^3, R is the gas constant, and T is the crustal temperature in Kelvin.

In Figure S4 we compare the variability and temporal trends of eruptible magma chemistry to the temperature dependent rheological evolution of overlying crustal rocks represented by the Deborah Number ($De \sim t_{inject}/t_{relax}$). This dimensionless number quantifies the range of elastically ($De >> 1$) to viscously ($De << 1$) dominated responses of crustal rocks as a ratio of 2 timescales (Karlstrom et al., 2007). A high magma flux system growing with

0.0178 km^3/yr (accretion rate = 0.052 m/yr) at crustal depth greater 20 km and initial geothermal gradient of 20°C/km is shown in Figure S4a. Similar to the scenario shown in figure 3a, the early systems is composed mostly of high silica liquids with short phases of basaltic magma invasion, yielding a large compositional variance, which drops while the reservoir matures towards more mafic chemistry. The time evolution of De is characterized by a progressively more viscous rheology of the overburden crustal rocks, indicating that magma storage is favoured over extraction in later stages of the systems lifecycle. Referring back to figure 3a, the large probability of occurrence for a multitude of compositions that characterize the late evolution of a high magma flux system, may lead to more homogenous erupted products in a regime dominated by storage and magma stirring (Huber et al., 2009; Karakas et al., 2017). Still, the potential to sample a range of different compositions through time for a high magma flux case is much larger compared to a reservoir built in hotter and deeper crust (>25 km depth, initial geothermal gradient of 30°C/km) at a low volumetric magma influx of 0.00056 km^3/yr, such as shown in Figure S4b. The chemical trend in such a case develops to only slightly more mafic compositions in time with limited variance, as a result of damped heating and cooling cycles and thermal buffering of the surrounding crustal rocks. The monotonous evolution of such a system, and its potential expression in volcanic rocks at the surface, is also reinforced by the high De viscous response of crustal rocks, favouring magma storage and homogenization.

Table S1: Thermal modelling parameters

List of parameters used in thermal modeling.	
Specific heat	1000 J kg^{-1} K^{-1}
Thermal conductivity	T dependent (Whittington et al., 2009)
Latent heat	3.13 x 10^5 J kg^{-1}
Density (melt and solid)	2700 kg m^{-3}
Initial geothermal gradient	25 °C km^{-1}
Surface temperature	8 °C
Initial intrusion depth	20 or 25 km
$T_{solidus}$	650 °C
$T_{liquidus}$	1170 °C

Table S2: Magma chemistry as a function of temperature adapted from Nandedkar et al., (2014). All temperatures are given in °C. F denotes melt fraction.

T interval	700-750	750-800	800-850	850-900	900-950	950-1000	1000-1050	1050-1100	1100-1150	1150-1170
SiO_2 (wt.%)	77.42	74.78	72.51	69.76	65.22	60.16	53.59	50.78	50.64	50.66
Al_2O_3	13.67	15.08	16.29	17.12	18.09	19.29	19.78	18.64	15.96	15.31
TiO_2	0.06	0.10	0.15	0.28	0.55	0.63	0.88	0.87	0.74	0.73
FeO	0.52	0.77	1.08	1.84	3.23	4.70	8.41	8.36	8.83	8.78
MgO	0.30	0.58	0.92	1.19	1.63	2.85	4.74	7.23	9.04	9.82
CaO	1.03	1.82	2.92	3.73	5.17	7.32	8.31	10.82	12.23	12.24
K_2O	2.47	2.39	2.14	2.05	1.77	1.34	0.98	0.77	0.67	0.64
Na_2O	4.35	4.23	3.63	3.61	3.90	3.29	2.94	2.19	1.97	1.92
Average F	0.18	0.22	0.25	0.27	0.35	0.40	0.50	0.7	0.87	1.00
Average T	725	775	825	875	925	975	1025	1075	1125	1160

Table S3: Representative major element analysis (XRF) for Nevado de Toluca (Mexico) whole rock samples.

Sample #	Stage*	SiO_2 (wt. %)	TiO_2 (wt. %)	Al_2O_3 (wt. %)	Fe_2O_3 (wt. %)	MnO (wt. %)	MgO (wt. %)	CaO (wt. %)	Na_2O (wt. %)	K_2O (wt. %)	P_2O_5 (wt. %)	LOI (wt. %)	Total
GW-16-01	5	65.59	0.65	16.9	4.13	0.07	1.74	4.16	4.43	2.04	0.14	0.79	99.86
GW-16-01gp	5	65.86	0.61	16.93	3.97	0.07	1.74	4.05	4.49	2.09	0.14	1.29	99.95
GW-16-01gp	5	65.73	0.61	16.89	3.98	0.07	1.74	4.06	4.49	2.09	0.14	1.31	99.79
GW-16-01wr	5	63.64	0.68	17.96	4.51	0.07	2.09	4.71	4.73	1.78	0.16	1.48	100.34
GW-16-01wr	5	63.3	0.68	17.92	4.48	0.07	2.08	4.7	4.51	1.77	0.16	1.54	99.68
GW-16-02	5	65.78	0.59	17.12	3.86	0.07	1.76	4	4.36	1.81	0.19	1.84	99.54
GW-16-08a	5	63.92	0.67	17.64	4.33	0.07	1.88	4.41	4.45	1.82	0.18	2.09	99.38
GW-16-08b	5	63.09	0.7	18.62	4.5	0.08	1.93	4.57	4.37	1.7	0.18	2.49	99.75
GW-16-08cgp	5	65.5	0.64	16.58	4.14	0.07	1.82	4.17	4.65	1.97	0.17	0.77	99.71
GW-16-08cwp	5	61.27	0.79	20.08	5.02	0.08	2.11	4.66	4.21	1.52	0.2	3.42	99.95
GW-16-09	5	65.82	0.62	16.44	4.06	0.07	1.81	4.18	4.48	2.05	0.17	1.48	99.71
GW-16-10	5	65.75	0.6	17.53	3.94	0.07	1.81	3.9	4.32	1.76	0.21	1.95	99.89
GW-16-13	4	65.03	0.51	17.89	3.42	0.06	1.49	4.83	4.92	1.7	0.14	0.16	99.99
GW-16-13b	4	63.5	0.67	18.24	4.38	0.07	1.94	4.35	4.36	1.86	0.22	2.75	99.59
GW-16-17	3-4	61.74	0.76	19.67	4.82	0.07	1.99	4.5	4.22	1.56	0.23	4.46	99.56
GW-16-20	5	64.6	0.58	16.55	4.24	0.07	2.51	4.9	4.12	1.85	0.14	2.80	99.58
GW-16-21	5	64.85	0.6	17.12	3.99	0.07	1.94	4.84	4.51	1.76	0.14	1.86	99.84
GW-16-23gp	5	65.25	0.61	16.43	4.09	0.07	1.99	4.4	4.48	2.1	0.15	1.42	99.59
GW-16-28	5	60.52	0.74	20.24	5.06	0.08	2.22	5.06	4.13	1.22	0.09	3.11	99.37
GW-16-28b	5	60.56	0.74	20.22	5.08	0.08	2.22	5.06	4.14	1.22	0.09	3.18	99.42
GW-16-28c	5	61.11	0.74	20.07	5.01	0.07	2.15	4.82	4.09	1.31	0.09	3.59	99.46
GW-16-29	5	65.22	0.61	16.88	4.14	0.07	1.95	4.38	4.17	1.88	0.15	2.38	99.45
GW-16-30	5	65.2	0.6	16.99	3.97	0.07	1.85	4.46	4.62	1.89	0.15	0.77	99.79
GW-16-31	5	65.74	0.58	16.87	3.81	0.07	1.68	4.37	4.73	1.89	0.15	0.51	99.89
GW-16-32	5	65.59	0.58	16.66	3.92	0.07	1.98	4.6	4.46	1.89	0.15	1.92	99.90
GW-16-33	5	65.59	0.6	17.36	3.94	0.07	1.66	4.07	4.13	1.95	0.16	3.55	99.53
LP-16-01	5	66.47	0.49	16.87	3.2	0.06	1.43	4.15	4.75	2.01	0.13	0.12	99.56
LP-16-04	5	66.05	0.49	17.2	3.35	0.06	1.56	4.34	4.71	1.97	0.13	2.71	99.87
LP-16-06	5	65.31	0.57	17.28	3.75	0.06	1.68	4.57	4.8	1.84	0.15	0.45	100.03
LP-16-06b	5	65.26	0.57	17.28	3.76	0.06	1.68	4.57	4.81	1.85	0.15	0.28	99.99
LP-16-10	4	64.61	0.54	17.68	3.61	0.06	1.61	4.77	4.86	1.69	0.15	0.41	99.61
LP-16-11	pre	61.11	0.71	16.55	5.37	0.09	3.9	6.09	4	1.83	0.17	0.37	99.84
LP-16-13	1	60.32	0.68	17.46	5.14	0.08	3.2	6.26	4.4	2.01	0.22	0.06	99.79
LP-16-14	3	63.04	0.49	18.11	3.61	0.06	3.33	4.75	4.74	1.71	0.04	1.27	99.91
LP-16-20	1	63.64	0.61	17.29	4.29	0.08	2.62	4.69	4.48	1.87	0.16	0.76	99.78
LP-16-21wr	3	65.77	0.55	16.87	3.72	0.06	1.65	4.36	4.63	2	0.13	0.38	99.75
LP-16-22	1	61.05	0.69	19.11	4.75	0.08	2.3	5.63	4.67	1.5	0.14	0.96	99.92
LP-16-23	5	62.78	0.56	17.69	4.07	0.07	2.51	5.56	4.67	1.66	0.15	0.96	99.73
LP-16-26	4	65.96	0.56	16.85	3.41	0.06	1.9	4.34	4.36	2.2	0.13	1.88	99.78
LP-16-29		58.94	0.84	16.69	7	0.11	4.95	5.71	3.89	1.47	0.16	0.62	99.79

* Eruptive stages were adapted from Torres-Orozco et al., (2017): stage 5 (3-57 ka), stage 4 (130-430 ka), stage 3 (0.86-1.2 Ma), stage 2 (1.1-1.3 Ma), stage 1 (1.25-1.5 Ma), pre Nevado (>3 Ma).

Table S4: Compilation of magma diversity, long-term eruption rates and volumes.

Volcanic System	Arc Segment	Crustal Thickness (km)	Median SiO_2 (wt. %)	2σ SiO_2 (wt. %)	Timespan (ka)	Volume (km^3)	Eruptive flux (km^3/ka)	Reference
Nevado de Toluca	Trans-Mexican	45-50	65.03	4.00	1500	60	0.04	This study; Torres-Orozco et al. 2017; Arce et al. 2006
Puyehue-Cordón Caulle	Andes SVZ	35–40	56.14	14.07	314	131	0.42	Singer et al. 2008
Tatara-San Pedro	Andes SVZ	30–35	55.28	9.56	930	55	0.06	Singer et al. 1997
Parinacota[1]	Andes CVZ	~70	61.42	8.77	163	46	0.28	Hora et al. 2007
Mt Adams Field[2]	Cascades	40–45	57.60	10.74	940	231	0.25	Hildreth and Lanphere 1994; Jicha et al. 2009
Mt Baker Field[3]	Cascades	40–45	60.18	9.90	1283	217	0.17	Hildreth et al., 2003
Mt Baker and Black Buttes[4]	Cascades	40–45	59.59	6.20	400	39	0.10	Hildreth et al., 2003
Mount Mazama	Cascades	40–45	62.68	9.53	420	176	0.42	Bacon and Lanphere 2006
Katmai cluster	Aleutians	30–36	61.20	9.82	292	210	0.72	Hildreth and Fierstein 2000
Seguam	Aleutians	30–35	58.64	12.48	318	79	0.25	Jicha and Singer 2006
Santorini	Aegean	20–32	59.65	11.99	650	300	0.46	Druitt et al. 1999
Soufrière Hills	Antilles	30–40	59.08	6.44	174	26	0.15	Harford et al. 2002
Aucanquilcha	Andes CVZ	~70	64.83	2.36	1000	38	0.04	Klemetti and Grunder 2008
El Chichon	Chiapanecan	25-30	56.53	2.82	372	26	0.07	Layer et al. 2009; Arce et al., 2015
Uturuncu	Andes CVZ	~70	64.40	2.72	800	50	0.06	Muir et al. 2015
Paniri[5]	Andes CVZ	~70	65.07	8.26	376	33	0.09	Godoy et al. 2018
Volcan Ceboruco	Trans-Mexican	35–40	56.4	12.12	100	51	0.51	Frey et al. 2004
Ceboruco-San Pedro[6]	Trans-Mexican	35–40	59.1	12.87	656	81	0.12	Frey et al. 2004
South Sister[7]	Cascades	40–45	64.47	10.46	49	18	0.37	Fierstein et al. 2011
Mt Griggs	Aleutians	30–36	58.14	4.39	290	25	0.09	Hildreth and Fierstein 2000
Trident volcano	Aleutians	30–36	61.66	6.03	143	22	0.15	Hildreth and Fierstein 2000

[1] Cone building phase at eruptive flux 0.92 km^3/ka with high diversity after lull in activity of ~60ka.
[2] Volume estimate between 231 and 400 for whole field. The andesitic cone was build at a rate of 1.5 in 3 spurts.
[3] Volume estimate 105-217 km^3 for whole field including caldera suite
[4] Baker stratocone and domes in vicinity without earlier caldera suite
[5] Not including the earlier Plateau shield stage due to long hiatus of ~750ka
[6] Entire cluster including Amado Nervo, pre 656 ka volume negligible
[7] Main buildup over about 15 ka at high rate

Table S5: Support for the importance of mid-deep crustal processes for various volcanic systems considered in this study.

Volcanic System	Support for mid-deep crustal reservoirs	Reference
Nevado de Toluca	Plagioclas chemistry	Smith et al. 2009
Tatara-San Pedro	Lava geochemistry	Feeley et al., 1998
Parinacota	Plagioclase chemistry	Ginibre and Wörner, 2007
Mt Adams Field	S wave anomaly	Flinders and Shen, 2017
Mount Mazama	U-Th isotopes	Ankney et al. 2013
Katmai cluster	S-wave shadow	Matumoto 1971
Santorini	Lava geochemistry	Mortazavi and Sparks, 2004
Soufrière Hills	Surface deformation	Elsworth et al. 2008
Aucanquilcha	Pyroxene barometry	Walker et al. 2013
Uturuncu	Surface deformation	Sparks et al. 2008
Trident volcano	Plagioclase textures	Coombs et al. 2000

References

Ankney, M. E., Johnson, C. M., Bacon, C. R., Beard, B. L., & Jicha, B. R. (2013). Distinguishing lower and upper crustal processes in magmas erupted during the buildup to the 7.7 ka climactic eruption of Mount Mazama, Crater Lake, Oregon, using 238 U–230 Th disequilibria. *Contributions to Mineralogy and Petrology*, *166*(2), 563-585.

Arce, J. L., Macias, J. L., Gardner, J. E., & Layer, P. W. (2006). A 2.5 ka history of dacitic magmatism at Nevado de Toluca, Mexico: petrological, 40Ar/39Ar dating, and experimental constraints on petrogenesis. *Journal of petrology*, *47*(3), 457-479.

Arce, J. L., Walker, J., & Keppie, J. D. (2015). Petrology and geochemistry of El Chichón and Tacaná: two active, yet contrasting Mexican volcanoes. In *Active Volcanoes of Chiapas (Mexico): El Chichón and Tacaná* (pp. 25-43). Springer, Berlin, Heidelberg.

Bacon, C. R., & Lanphere, M. A. (2006). Eruptive history and geochronology of Mount Mazama and the Crater Lake region, Oregon. *Geological Society of America Bulletin*, *118*(11-12), 1331-1359.

Coombs, M. L., Eichelberger, J. C., & Rutherford, M. J. (2000). Magma storage and mixing conditions for the 1953–1974 eruptions of Southwest Trident volcano, Katmai National Park, Alaska. *Contributions to Mineralogy and Petrology*, *140*(1), 99-118.

Druitt, T. H., Edwards, L., Mellors, R. M., Pyle, D. M., Sparks, R. S. J., Lanphere, M., ... & Barreirio, B. (1999). Santorini volcano. *Geological Society Memoir*, *19*.

Elsworth, D., Mattioli, G., Taron, J., Voight, B., & Herd, R. (2008). Implications of magma transfer between multiple reservoirs on eruption cycling. *Science*, *322*(5899), 246-248.

Feeley, T. C., Dungan, M. A., & Frey, F. A. (1998). Geochemical constraints on the origin of mafic and silicic magmas at Cordon El Guadal, Tatara-San Pedro Complex, central Chile. *Contributions to Mineralogy and Petrology*, *131*(4), 393-411.

Fierstein, J., Hildreth, W., & Calvert, A. T. (2011). Eruptive history of South Sister, Oregon Cascades. *Journal of Volcanology and Geothermal Research*, *207*(3-4), 145-179.

Flinders, A. F., & Shen, Y. (2017). Seismic evidence for a possible deep crustal hot zone beneath Southwest Washington. *Scientific reports*, *7*(1), 7400.

Frey, H. M., Lange, R. A., Hall, C. M., & Delgado-Granados, H. (2004). Magma eruption rates constrained by 40Ar/39Ar chronology and GIS for the Ceboruco–San Pedro volcanic field, western Mexico. *Geological Society of America Bulletin*, *116*(3-4), 259-276.

Ginibre, C., & Wörner, G. (2007). Variable parent magmas and recharge regimes of the Parinacota magma system (N. Chile) revealed by Fe, Mg and Sr zoning in plagioclase. *Lithos*, *98*(1-4), 118-140.

Godoy, B., Lazcano, J., Rodríguez, I., Martínez, P., Parada, M. A., Le Roux, P., ... & Polanco, E. (2018). Geological evolution of Paniri volcano, Central Andes, northern Chile. *Journal of South American Earth Sciences*, *84*, 184-200.

Harford, C. L., Pringle, M. S., Sparks, R. S. J., & Young, S. R. (2002). The volcanic evolution of Montserrat using 40Ar/39Ar geochronology. *Geological Society, London, Memoirs*, *21*(1), 93-113.

Hildreth, W., & Lanphere, M. A. (1994). Potassium-argon geochronology of a basalt-andesite-dacite arc system: The Mount Adams volcanic field, Cascade Range of southern Washington. *Geological Society of America Bulletin*, *106*(11), 1413-1429.

Hildreth, W., & Fierstein, J. (2000). Katmai volcanic cluster and the great eruption of 1912. *GSA Bulletin*, *112*(10), 1594-1620.

Hildreth, W., Fierstein, J., & Lanphere, M. (2003). Eruptive history and geochronology of the Mount Baker volcanic field, Washington. *Geological Society of America Bulletin*, *115*(6), 729-764.

Hora, J. M., Singer, B. S., & Wörner, G. (2007). Volcano evolution and eruptive flux on the thick crust of the Andean Central Volcanic Zone: 40Ar/39Ar constraints from Volcán Parinacota, Chile. *GSA Bulletin*, *119*(3-4), 343-362.

Huber, C., Bachmann, O., & Manga, M. (2009). Homogenization processes in silicic magma chambers by stirring and mushification (latent heat buffering). *Earth and Planetary Science Letters*, *283*(1-4), 38-47.

Jicha, B. R., & Singer, B. S. (2006). Volcanic history and magmatic evolution of Seguam Island, Aleutian Island arc, Alaska. *Geological Society of America Bulletin*, *118*(7-8), 805-822.

Jicha, B. R., Hart, G. L., Johnson, C. M., Hildreth, W., Beard, B. L., Shirey, S. B., & Valley, J. W. (2009). Isotopic and trace element constraints on the petrogenesis of lavas from the Mount Adams volcanic field, Washington. *Contributions to Mineralogy and Petrology*, *157*(2), 189-207.

Jicha, B. R., Laabs, B. J., Hora, J. M., Singer, B. S., & Caffee, M. W. (2015). Early Holocene collapse of Volcán Parinacota, central Andes, Chile: Volcanological and paleohydrological consequences. *Bulletin*, *127*(11-12), 1681-1688.

Karakas, O., Degruyter, W., Bachmann, O., & Dufek, J. (2017). Lifetime and size of shallow magma bodies controlled by crustal-scale magmatism. *Nature Geoscience*, *10*(6), 446-450.

Karlstrom, L., Paterson, S. R., & Jellinek, A. M. (2017). A reverse energy cascade for crustal magma transport. *Nature Geoscience*, *10*(8), 604-608.

Klemetti, E. W., & Grunder, A. L. (2008). Volcanic evolution of Volcán Aucanquilcha: A long-lived dacite volcano in the Central Andes of northern Chile. *Bulletin of Volcanology*, *70*(5), 633-650.

Layer, P. W., García-Palomo, A., Jones, D., Macías, J. L., Arce, J. L., & Mora, J. C. (2009). El Chichón volcanic complex, Chiapas, México: Stages of evolution based on field mapping and 40Ar/39Ar geochronology. *Geofísica internacional*, *48*(1), 33-54.

Matumoto, T. (1971). Seismic body waves observed in the vicinity of Mount Katmai, Alaska, and evidence for the existence of molten chambers. *Geological Society of America Bulletin*, *82*(10), 2905-2920.

Mortazavi, M., & Sparks, R. S. J. (2004). Origin of rhyolite and rhyodacite lavas and associated mafic inclusions of Cape Akrotiri, Santorini: the role of wet basalt in generating calcalkaline silicic magmas. *Contributions to Mineralogy and Petrology*, *146*(4), 397-413.

Muir, D. D., Barfod, D. N., Blundy, J. D., Rust, A. C., Sparks, R. S. J., & Clarke, K. M. (2015). The temporal record of magmatism at Cerro Uturuncu, Bolivian Altiplano. *Geological Society, London, Special Publications*, *422*(1), 57-83.

Nandedkar, R. H., Ulmer, P., & Müntener, O. (2014). Fractional crystallization of primitive, hydrous arc magmas: an experimental study at 0.7 GPa. *Contributions to Mineralogy and Petrology*, *167*(6), 1015.

Torres-Orozco, R., Arce, J. L., Layer, P. W., & Benowitz, J. A. (2017). The Quaternary history of effusive volcanism of the Nevado de Toluca área, Central México. *Journal of South American Earth Sciences*, *79*, 12-39.

Singer, B. S., Thompson, R. A., Dungan, M. A., Feeley, T. C., Nelson, S. T., Pickens, J. C., ... & Metzger, J. (1997). Volcanism and erosion during the past 930 ky at the Tatara–San Pedro complex, Chilean Andes. *Geological Society of America Bulletin*, *109*(2), 127-142.

Singer, B. S., Jicha, B. R., Harper, M. A., Naranjo, J. A., Lara, L. E., & Moreno-Roa, H. (2008). Eruptive history, geochronology, and magmatic evolution of the Puyehue-Cordón Caulle volcanic complex, Chile. *Geological Society of America Bulletin*, *120*(5-6), 599-618.

Smith, V. C., Blundy, J. D., & Arce, J. L. (2009). A temporal record of magma accumulation and evolution beneath Nevado de Toluca, Mexico, preserved in plagioclase phenocrysts. *Journal of Petrology*, *50*(3), 405-426.

Sparks, R. S. J., Folkes, C. B., Humphreys, M. C., Barfod, D. N., Clavero, J., Sunagua, M. C., ... & Pritchard, M. E. (2008). Uturuncu volcano, Bolivia: Volcanic unrest due to mid-crustal magma intrusion. *American Journal of Science*, *308*(6), 727-769.

Walker, B. A., Klemetti, E. W., Grunder, A. L., Dilles, J. H., Tepley, F. J., & Giles, D. (2013). Crystal reaming during the assembly, maturation, and waning of an eleven-million-year crustal magma cycle: thermobarometry of the Aucanquilcha Volcanic Cluster. *Contributions to Mineralogy and Petrology, 165*(4), 663-682.

Whittington, A. G., Hofmeister, A. M., & Nabelek, P. I. (2009). Temperature-dependent thermal diffusivity of the Earth's crust and implications for magmatism. *Nature*, *458*(7236), 319-321.
